# Microstructural Characterization and Property of Carbon Fiber Reinforced High-Density Polyethylene Composites Fabricated by Fused Deposition Modeling

**DOI:** 10.3390/ma16010180

**Published:** 2022-12-25

**Authors:** Partha Pratim Pandit, Chang Liu, Scott Iacono, Giancarlo Corti, Yingbin Hu

**Affiliations:** 1 Department of Mechanical and Manufacturing Engineering, Miami University, Oxford, OH 45056, USA; 2 Chemistry Department and Chemistry Research Center, United States Air Force Academy, Colorado Springs, CO 80840, USA

**Keywords:** fused deposition modeling, carbon fiber-reinforced high-density polyethylene, microstructure characterization, tensile properties, dynamic mechanical analysis, thermogravimetric analysis

## Abstract

As a promising industrial thermoplastic polymer material, high-density polyethylene (HDPE) possesses distinct properties of ease to process, good biocompatibility, high recyclability, etc. and has been widely used to make packaging, prostheses and implants, and liquid-permeable membranes. Traditional manufacturing processes for HDPE, including injection molding, thermoforming, and rotational molding, require molds or post processing. In addition, part shapes are highly restricted., Thus, fused deposition modeling (FDM) is introduced to process HDPE materials to take advantage of FDM’s free of design, no mold requirement, ease and low cost of processing. To improve the mechanical properties (such as stiffness and strength) and thermal resistance of HDPE, carbon fiber (CF) was incorporated into HDPE, and CF-reinforced HDPE composites were successfully fabricated using FDM process. In addition, the effects of CF content on surface quality, microstructure characterizations, tensile properties, dynamic mechanical properties, and thermal properties have been investigated. Experimental results show that an appropriate CF content addition is beneficial for improving surface quality, and mechanical and thermal properties.

## 1. Introduction

Owing to their indisputable chemical stability, optical transparency, durability, self-lubrication ability, and hydrophobic or waterproofing potential, thermoplastic polymers are finding their ways into various research and industry applications [1]. The aerospace, biomedical, and automotive industries have already become early adopters of thermoplastic polymers for manufacturing intermediate tooling and even end-use parts, in addition to prototyping. Besides the above-mentioned properties, simplicity of processing and bio-inert characteristics are two major reasons why the thermoplastic polymers are widely used in these industries [2]. High-density polyethylene (HDPE), as an important semi-crystalline thermoplastic polymer, is considered as a promising industrial thermoplastic polymer that has been utilized to make prostheses and implants, liquid-permeable membranes, corrosion-resistant pipes, packaging, and a variety of other functional parts [3], owing to its distinct physical and mechanical properties, such as easy and low cost of processing, good biocompatibility, high recyclability, excellent abrasion and fatigue behavior, and high impact resistance [4,5].

Like other thermoplastic polymers, melt-and-form/reform manufacturing techniques (e.g., injection molding, thermoforming, rotational molding, etc.) have traditionally been used to process HDPE [6]. Injection molding involves steps of melting the polymer, filling the molten polymer into the mold cavity, and cooling. During the cooling step, volume shrinkage may result in warping of the final product [7]. The semi-crystalline characteristic of HDPE makes it shrink more than other amorphous polymers do [8]. Traditional manufacturing processes also encounter other problems (such as restricted shapes and requirements of mold or post processing) when processing HDPE materials. The shape restriction and mold requirement problems can be addressed by additive manufacturing (AM), which is a process of joining materials layer-upon-layer following a three-dimensional digital model, unlike the traditional subtractive or formative manufacturing processes [9]. The ASTM classifies AM technologies into seven primary categories, materials extrusion, material jetting, vat photopolymerization, binder jetting, powder bed fusion, directed energy deposition, and sheet lamination [10]. Fused deposition modeling (FDM) is a material extrusion-based economic AM technology designed to process polymer and composite materials, and it has been widely used in aerospace, biomedical, and packaging industries to produce products [11]. The advantages of FDM lie in the low cost, short fabrication time, and ease of handling [12].

**Figure 1:**
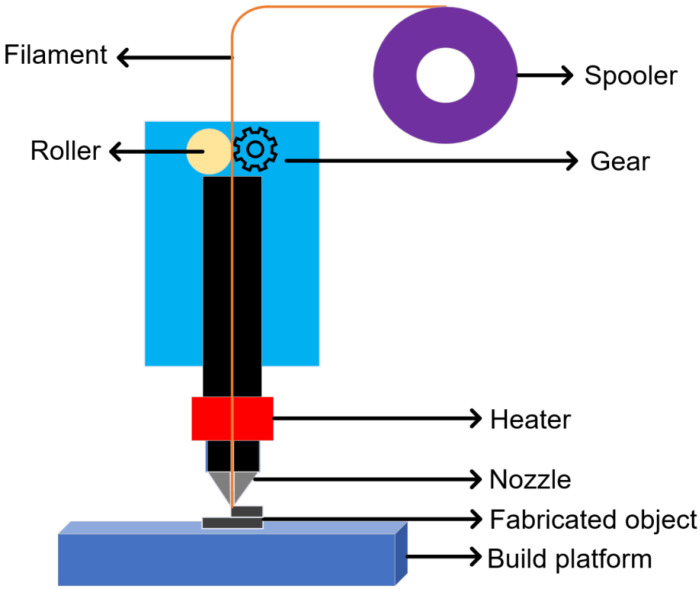
A schematic of the FDM process.

Figure 1 shows a schematic of a typical FDM process. In the FDM process, filaments are fed through the gap between a roller and a gear to the deposition head, where HDPE filaments are heated and melted, then deposited onto the build platform. Once extruded and deposited onto the build platform, the semi-solid plastic material solidifies quickly. The printing nozzle moves in the horizontal x and y directions, whereas the build platform moves in the vertical z-direction. The printing nozzle moves along the trajectory designed by the 3D digital model to create one layer. Afterward, the platform is lowered in the z-direction prior to the printing of another new layer. This process continues until the whole object is constructed.

Despite the above-mentioned benefits and ongoing studies, HDPE is yet to become prominent in FDM due to several issues. Firstly, HDPE can hardly be deposited onto any materials but hot HDPE, making the selection of build platform limited. Detaching HDPE parts from a hot HDPE build platform after printing can also be problematic. Secondly, volume shrinkage upon cooling makes it challenging to maintain dimensional accuracy. Thirdly, HDPE suffers from warping during a rapid cooling process, which further weakens the adherence of printed parts to the build platform, causing interference problems between printed parts and the printing nozzle [13]. When it comes to shrinkage, Spoerk and Sapkota discovered that adding spherical fillers can help reduce shrinkage by increasing tensile strength and toughness, which is useful for shrinkage reduction [14]. Fibers are also found to be effective in reducing shrinkage. Chinga-Carrasco et al. effectively printed two types of bio-based HDPE composites using FDM after adding thermomechanical pulp fibers to the HDPE matrix [15]. The warpage issue can be mitigated by optimizing process parameters. The optimized process parameter can also improve mechanical properties [16].

Even though some of the aforementioned printing concerns have been addressed, parts made from HDPE materials still exhibit poor properties due to HDPE’s inherent low mechanical properties and thermal resistance. Therefore, it is critical to enhance FDM-printed HDPE’s properties by incorporating reinforcements, such as carbon fiber (CF), silica particles, and glass fiber. Among all of these reinforcement materials, CF was selected due to CF’s high strength-to-weight ratio, recycling capability, and enhanced thermal properties [17,18,19]. In addition, CF-reinforced thermoplastic composites are being reported as potential materials to replace traditional thermoplastic polymers and even metals [20]. In a previous investigation, CF concentrations of up to 4 wt.% were considered to investigate its effect on mechanical and dynamic mechanical properties [21]. In the present project, the effects of CF content, with a concentration up to 14%, on surface quality, microstructure characterizations, tensile properties, dynamic mechanical properties, and thermal properties have been analyzed for the first time.

## 2. Experiments and Measurement Procedures

### 2.1. Material and Filament Preparation

**Figure 2:**
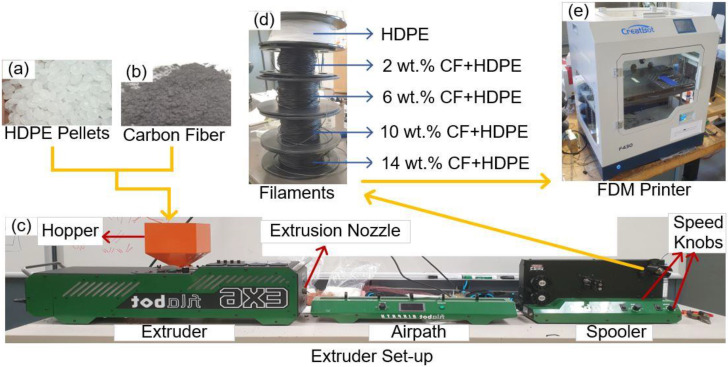
Experimental setup: filament preparation and FDM process. (**a**) HEPE pellets, (**b**) Carbon fiber, (**c**) extruder set-up, (**d**) filaments of CF+HDPE with different CF contents, and (**e**) a picture of the PDM printer used in this investigation.

In this study, five different types of filaments, including pure HDPE, 2 wt.% CF + HDPE, 6 wt.% CF + HDPE, 10 wt.% CF + HDPE, and 14 wt.% CF + HDPE are prepared using an extruder (EX6, Filabot, Barre, VT, USA). Figure 2a–d show the materials and experimental setup used for filament preparation. The HDPE (VViViD Vinyls, Quebec, Canada) is in the form of pellets with an average size of 5 mm. Micro-sized CFs are sourced from the Zoltek Corporation (St. Charles, MO, USA). Key features of the as-received CF are listed in Table 1. Prior to making CF + HDPE filaments, a mixture between CF and HDPE was made using a magnetic stirrer (MS-500, Intllab, Biobase Biodustry, Jinan, China). The extruder fully melts the mixed materials by gradually increasing the temperature from the hopper to the extrusion nozzle. At the extrusion nozzle, the temperature is 150 °C, which is above the melting temperature of HDPE (125 °C). The airpath then cools the newly made filament right after it gets out of the extrusion nozzle and before it reaches the spooler. The diameter of the prepared filament is controlled by adjusting the speed of the spooler via using spooler speed knobs, as shown in Figure 2c. The actual diameter is constantly measured to timely change extrusion speed, thus making filaments with uniform cross-sections.

### 2.2. Experimental Setup and Conditions

**Figure 3:**
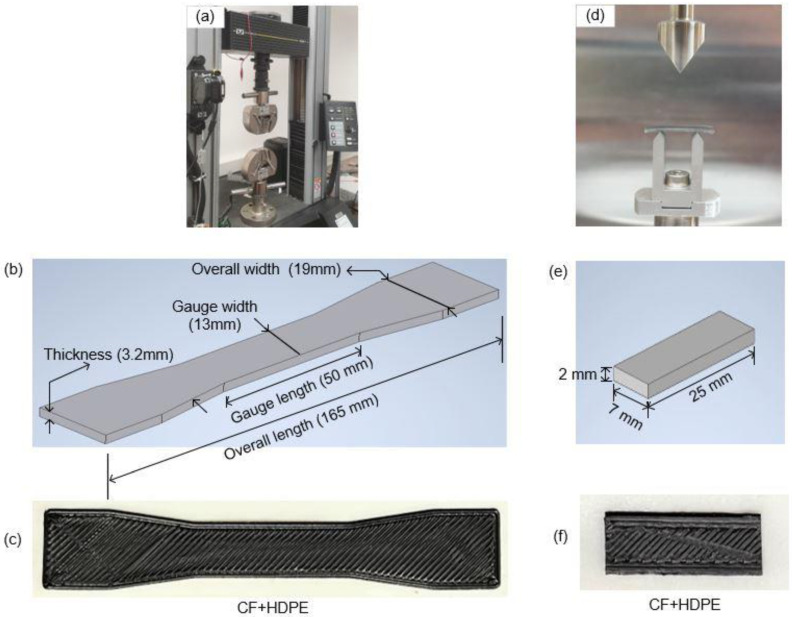
Experimental setup and specimens used to test mechanical properties: (**a**) a tensile test machine, (**b**) a schematic of a dog-bone part with dimensions for tensile tests, (**c**) a fabricated dog-bone part, (**d**) a DMA test machine, (**e**) a schematic of a rectangular part for DMA test, and (**f**) a fabricated part for DMA.

An FDM-based 3D printer (F430, Creatbot, Zhengzhou, Henan, China), as shown in Figure 2e, is used to perform the AM process. Table 2 lists process parameter values (or ranges) of the FDM process. Preliminary experiments were conducted to fabricate high-quality HDPE parts and CF-reinforced HDPE composites successfully. As aforementioned, special attention was given to controlling the speed of the spooler for making uniform cross-sectioned filaments with a diameter of 1.75 mm. In order to investigate the effects of CF on surface quality, microstructure characterization, tensile properties, and thermal properties, parts made from the five different types of filaments were prepared, including pure HDPE parts, and 2 wt.%, 6 wt.%, 10 wt.%, and 14 wt.% CF-reinforced HDPE composites. Dog-bone shapes for tensile tests were prepared according to the ASTM D638 Type I test specimen standard [22] Figure 3a–c illustrate a tensile test machine, a schematic of a dog-bone part, and a fabricated dog-bone part, respectively. Rectangular-shaped parts were also printed alongside dog bone shaped parts for dynamic mechanical analysis (DMA) testing. Figure 3d–f show a DMA machine, a schematic of a rectangular part, and a fabricated part for DMA testing.

### 2.3. Measurement Procedures

Calipers were used to measure the dimensions of the parts. Surface quality (such as surface roughness and surface morphology) was measured and observed using a stereo microscope (DSX1000, Olympus, Westborough, MA, USA). Microstructure characterizations of produced parts were characterized using a scanning electron microscopy (SEM) (Supra 35VP, Zeiss, Herbon, Kentucky, USA). SEM samples were cut from printed parts and then mounted in resin holders. Once the resin holders were cured, they were coated with gold to make the parts conductive prior to SEM analysis. A universal tensile tester (5867, Instron, Norwood, MA, USA) was used to conduct tensile tests at a fixed loading rate of 5 mm/min. Data were saved every 0.4 s for the time channel. To examine dynamic mechanical properties of HDPE and CF-reinforced HDPE composites, a DMA machine (RSA3, TA Instruments, New Castle, DE, USA) was used with a fixed frequency of oscillating stress and a dynamic strain of 1 Hz and 0.03%, respectively. With a constant ramping pace of 5 °C/min, the temperature was steadily increased from room temperature (23 °C) to 100 °C. Thermogravimetric analyses TGA (TGAQ500, TA Instruments, New Castle, DE, USA) were conducted under a nitrogen atmosphere up to 800 °C with a heating rate of 10 °C/min.

## 3. Results and Discussion

### 3.1. Effects on Surface Quality

**Figure 4:**
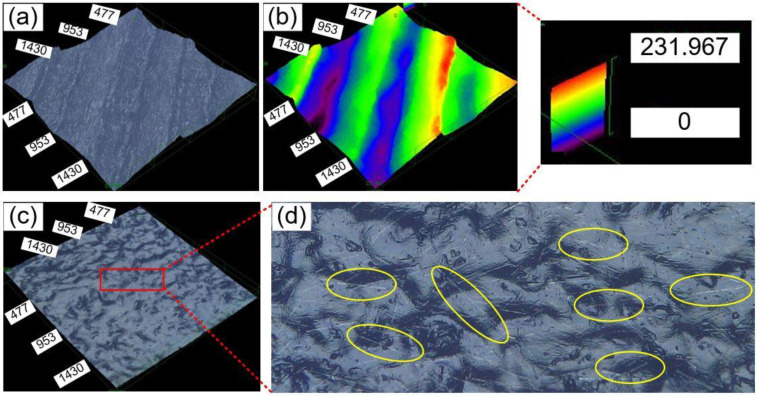
Surface morphology of a 6 wt.% CF-reinforced HDPE composite: (**a**) top surface, (**b**) height variation represented with different colors for the top surface, (**c**) bottom surface, and (**d**) a magnified area on the bottom surface with highlighted carbon fibers.

Experimental results show that CF contents have little impact on surface quality, including both surface roughness and surface morphology. The surface roughness of parts’ top surfaces ranges from 10 to 20 µm with no particular trend. Compared to top surfaces, the surface roughness of bottom surfaces (the surfaces directly attached to the build platform) of all parts is much smaller, ranging from 2 to 6 µm. Figure 4a shows a top surface morphology of a 6 wt.% CF-reinforced HDPE composite. The multiple tracks (with peaks and valleys) on the top surface indicate the printing trajectory of the part’s last layer. The height variation of the top surface is represented by different colors, among which red represents the largest height (value), and purple color represents the lowest height (value), as shown in Figure 4b. The height difference within this tested area is around 230 µm. Figure 4c shows the surface morphology of the bottom surface. Compared to the top surface, the bottom surface is flatter with a smaller height range. Figure 4d is a magnified area on the bottom surface and it demonstrates a great amount of CFs, which are embedded in the HDPE matrix.

### 3.2. Effects on Microstructure Characterizations

**Figure 5:**
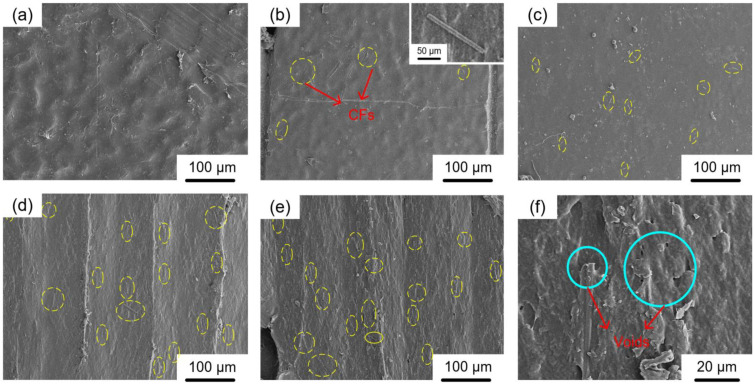
SEM images on cross-sections of parts with different CF contents: (**a**) a HDPE part, (**b**) a 2 wt.% CF + HDPE composite, (**c**) a 6 wt.% CF + HDPE composite, (**d**) a 10 wt.% CF + HDPE composite, (**e**) 14 wt.% CF + HDPE part, and (**f**) a 14 wt.% CF + HDPE part with magnified bonding area.

Figure 5a–e show microstructure characterizations of a pure HDPE part, a 2 wt.% CF-reinforced HDPE composite, a 6 wt.% CF-reinforced HDPE composite, a 10 wt.% CF-reinforced HDPE composite, and a 14 wt.% CF-reinforced HDPE composite, respectively. In these figures, CFs are circled by yellow-dashed lines. It can be seen from Figure 5a to Figure 5e that when CF content is increased from 0 wt.% to 14 wt.%, the number of circles increases with more CFs. In addition, CFs are uniformly distributed within HDPE matrix with no evident agglomeration. This proves that the HDPE and CF were well mixed with each other. In Figure 5b, a CF is magnified and shown in the right top corner. The length and diameter of this CF are 125 μm and 9 μm, respectively, matching the size and shape of the as-received CF (in Table 1) and indicating that the filament preparation and FDM printing process did not damage nor reshape CFs. For parts with CF contents ranging from 0 wt.% to 10 wt.%, no severe defects (such as noticeable delamination and micro-voids) are observed. When CF content is further increased to 14 wt.%, however, defects become more noticeable. Figure 5f shows a magnified figure of a certain area in Figure 5e. In Figure 5f, it can be seen that micro-voids are induced by introducing CFs and CFs partially delaminate from the HDPE matrix. The micro-voids and delamination problems are also found for other CF-reinforced HDPE composites. The higher the CF content is, the more micro-voids and more severe delamination problems the specimens presented. The reason behind this can be that with high CF concentration, the mixing between CF and HDPE matrix is not as good as with low CF concentration, since the high concentration has reached the saturation point of CF concentration [23].

### 3.3. Effects on Tensile Properties

**Figure 6:**
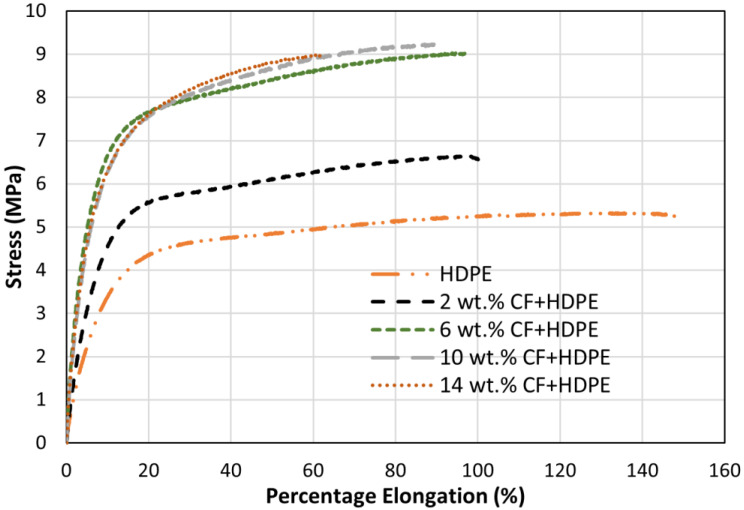
Effects of CFs on tensile properties.

Figure 6 shows the effects of CF content on tensile properties, and Table 3 summarises the tensile properties for the five types of parts. It can be observed from Figure 6 that tensile properties significantly vary with and without the incorporation of CF. For pure HDPE, the ultimate tensile stress (UTS) and Young’s moduli are 5.33 MPa and 95 MPa, respectively. With just a 2 wt.% CF incorporation, a dramatic 24% increase and a 53% increase are found for UTS and Young’s modulus. When adding more CFs (from 2 wt.% to 6 wt.%), the UTS and Young’s modulus are increased by 37% and 24%, respectively. Compared to 6 wt.% CF-reinforced HDPE composite, the UTS and Young’s modulus of 10 wt.% CF-reinforced HDPE further increase at a lower rate (2% for UTS and 22% for Young’s modulus). The increase in UTS and Young’s modulus induced by adding CF are in twofold. Firstly, CF possesses much higher strength (1000~7000 MPa) and Young’s modulus (50–950 GPa), compared to HDPE [24].

Secondly, when CF content is increased, the interparticle spacing among CFs is reduced, leading to increased resistance to dislocation motion and adding composites’ strength [25]. An increase of CF content from 10 wt.% to 14 wt.%, however, leads to a reduced UTS but Young’s modulus still increases. This can be attributed to the larger amount of CF number and micro-voids in the CF-reinforced HDPE composites (in Figure 5f), not present on samples with less than 10% of CF. Once it get pasts the yield point, these micro-voids act as stress raisers in the composites. More micro-voids mean higher stress concentration and amplification, which result in a faster fracture rate at lower applied stress. Furthermore, more CFs induce higher chances of delamination between CFs and the HDPE matrix, resulting in debonding and even considerable composite separation at low applied stress [25].

Although UTS and Young’s modulus have significant improvement by adding a certain amount of CF, an overall decrease in percentage elongation is observed when CF content is added and increased. By adding just 2 wt.% CF, the percentage elongation is dramatically reduced by 32%. Then, the decrease rate slowed down, and a 13% reduction is observed when increasing CF content from 2 wt.% to 10 wt.%. The reduced percentage elongation with increased CF content results from the inherited brittleness of CF. When increasing CF contents from 10 wt.% to 14 wt.%, a much larger reduction of 27% is observed for percentage elongation. The accelerated reduction rate could be attributed to the micro-voids and delamination generated in the 14 wt.% CF-reinforced HDPE composite.

Toughness is a measure of absorbed energy prior to the material fracturing failure. From Table 3, it can be concluded that adding a 2 wt.% of CF reduces toughness by 18%, compared to the pure HDPE part. The significant reduction can be attributed to the decreased percentage elongation induced by the CF. The toughness of 6 wt.% CF-reinforced composite is much higher than that of the 2 wt.% CF-reinforced composite is even higher than the pure HDPE part. The dramatic increase in stress-induced by 6 wt.% CF is the major reason for the improved toughness. When CF content is further increased from 6 wt.% to 14 wt.%, the toughness keeps decreasing.

### 3.4. Effects on DMA Properties

Materials that will be employed under severe dynamic loading and unloading working circumstances need to have good dynamic mechanical properties. The storage modulus ($E^{\prime }$), loss modulus ($E^{\prime \prime }$), and loss tangent (tanδ) can be expressed as [26]:

(1) $$E^{\prime }=\frac{\sigma_{0}}{\varepsilon_{0}}cos\delta$$

(2) $$E^{\prime \prime }=\frac{\sigma_{0}}{\varepsilon_{0}}sin\delta$$

(3) $$tan\delta =\frac{E^{\prime \prime }}{E^{\prime }}$$

where, $\sigma_{0}$ represents the maximum stress; $\varepsilon_{0}$ is the maximum amplitude of the strain; and δ represents phase lag.

**Figure 7:**
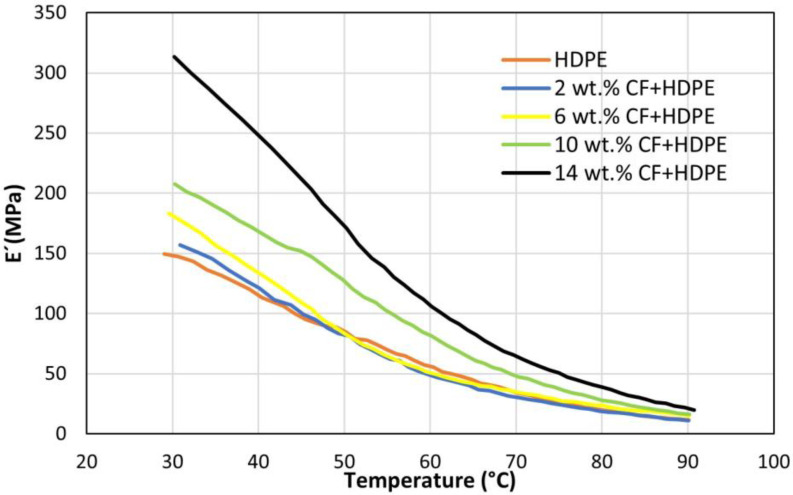
Effects of CFs on storage modulus (*E′*).

Figure 7 shows the effects of CF on storage moduli ($E^{\prime }$) of HDPE and CF-reinforced HDPE composites. With an increase in temperature, an overall decrease of storage moduli is observed for all the parts. This is due to the reason that the starting temperature (23 °C) is higher than the glass transition temperature (−135 °C) of HDPE [27]. It was reported that when the temperature is above the glass transition temperature, the storage modulus is decreased as the temperature increases [28]. This can be traced back to the fact that the increase in temperature leads to a decrease in molecular force, thus reducing the storage modulus. Another important conclusion that can be drawn from the figure is that at a corresponding temperature, the higher the CF content is, the higher the storage modulus when CF content is no greater than 14 wt.%. Thus, proving that adding CF to the HDPE matrix can significantly improve storage modulus. The major reason is that CF is much stiffer than HDPE. This aligns with the conclusion to Young’s modulus results of the same parts.

**Figure 8:**
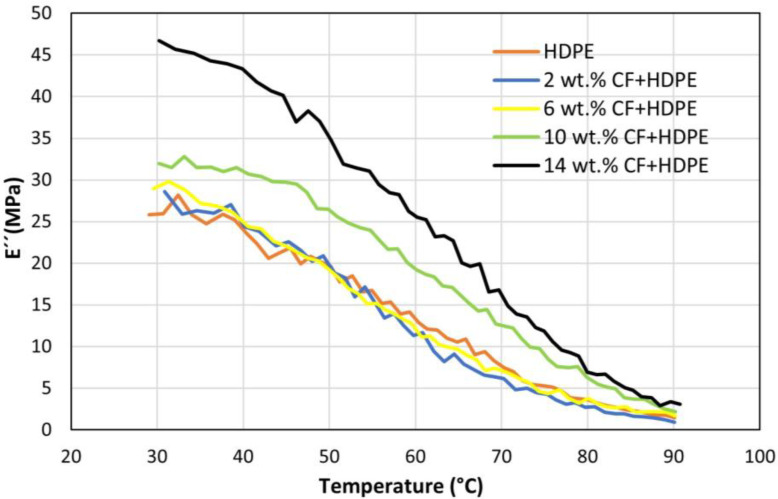
Effects of CFs on loss modulus (*E″*).

**Figure 9:**
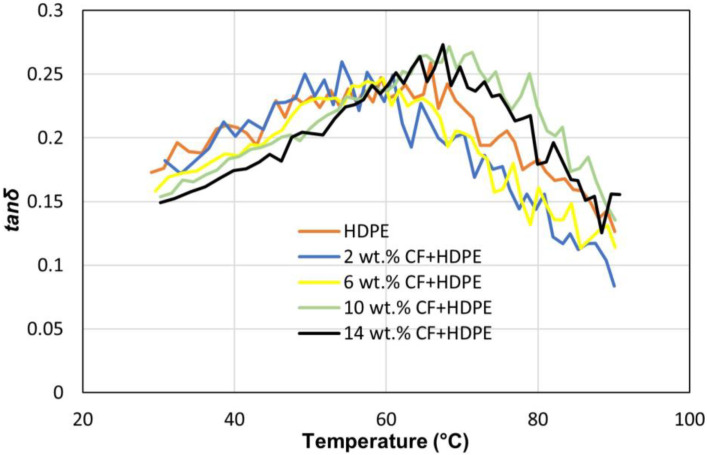
Effects of CFs on loss tangent (*tanδ*).

Figure 8 shows the effects of CF on loss modulus $\left(E^{\prime \prime }\right)$. Loss modulus represents the viscous portion of the material, and it measures energy dissipation upon heat. For all types of parts, their loss moduli are smaller than their storage moduli at the same temperature. Similar to the results for storage modulus, each part’s loss modulus also decreases with the increase of temperature within the testing range, conforming to the statement that when the temperature is above the glass transition temperature, the loss modulus also decreases with the increase in temperature [29]. This is attributed to the fact that when the temperature is over the glass transition of the material, the molecular friction of the material decreases and less energy is dissipated, resulting in a reduced loss modulus [29]. Similar to the results of the storage modulus, the loss modulus keeps increasing when CF content is increased from 0 wt.% to 14 wt.%. The reason behind this can be explained by the Kelvin-Voigt model, which shows that the part’s stiffness increases due to the addition of more and more CF into the HDPE matrix, inherently leading to a higher effective viscosity in the material [30]. It is understandable that while adding CF improves both storage modulus and Young’s modulus, the loss modulus will also increase. Because in the linear region of the stress strain curve, it would than dissipate more energy to get back to its original shape as the CF concentration increases. The loss tangent (tanδ) is a viscoelasticity index that is defined as a ratio between the loss modulus and the storage modulus. As shown in Figure 9, the loss tangent values of all parts are below 0.273, indicating that the parts act more elastically than plastically. The reason behind is that there is no reason why chains should have to use the same conformational pathway to get back to their equilibrium conformations; once the stress is eliminated, the material returns to its equilibrium shape. They are able to return to their initial position by taking a lower-energy path that has less resistance between nearby chains because they have moved from their original place. As long as this energy loss area is smaller than the region covered by the storage modulus curve, the polymer will behave elastically [31]. The loss tangent changing trends of all specimens are similar, and only slight differences exist among these parts, showing that the CF has little impact on loss tangent. When the temperature rises from 23 °C to 60 °C, the loss tangent also increases. One major reason for this phenomenon is that when the temperature rises, micro-voids or gaps between the CF reinforcement and the HDPE matrix expand, making the storage modulus drop more rapidly than the loss modulus does [32]. When the temperature is further increased, the dropping rate of loss modulus is higher than that of storage modulus, leading to the decrease of loss tangent. The ranges of all dynamic mechanical properties are summarized in Table 4.

### 3.5. Effects on Thermal Properties

**Figure 10:**
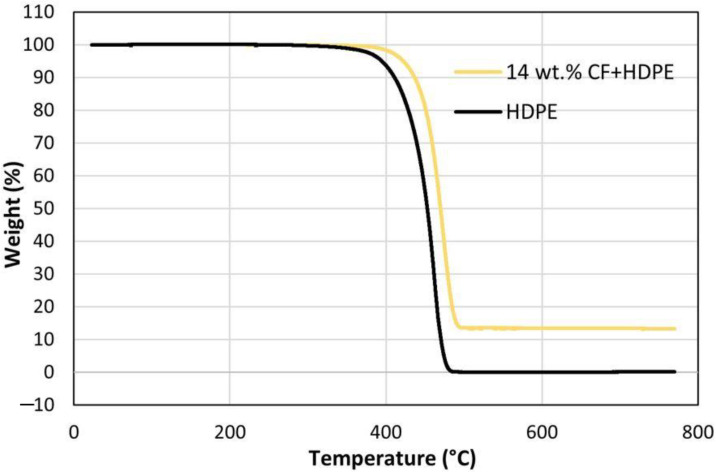
Effects of CFs on thermogravimetric properties.

Thermal stability and extent of degradation can be analyzed through weight loss studies, which use a temperature ramp method [33]. In this investigation, thermogravimetric analysis (TGA) was carried out to study the impact of CFs on the thermal stability and thermal degradation behaviors of the HDPE matrix. Figure 10 shows the percentage of remaining weight-as a function of temperature change for pure HDPE and 14 wt.% CF-reinforced HDPE materials. The 14 wt.% CF-reinforced HDPE composite has ~22 °C increased decomposition temperature than the pure HDPE, measured at the onset of the curve. This is attributed to CF reacting with low-molecular-weight alkyl radicals in the early stages of degradation (before 400 °C), the degradation of 14 wt.% CF-reinforced HDPE composite is slower than that of pure HDPE [34]. To summarize, an addition of CFs improves the thermal stability of HDPE matrix. The restriction of polymer chain mobility and exceptional thermal stability induced by the CF contributes to the improvement [35].

## 4. Conclusions

CF-reinforced HDPE composites with various CF contents were fabricated using the FDM process, and the effects of CF on surface quality, microstructure characterizations, mechanical properties (including tensile properties and DMA properties), and thermal properties were studied. Results show that CF content has little impact on surface quality. CFs were uniformly distributed within HDPE matrix with no apparent agglomeration, proving that the HDPE and CF were well mixed during filament preparation. No severe defects (such as noticeable delamination and micro-voids) were observed for CF content ranging from 0 wt.% to 10 wt.%. When CF content is further increased to 14 wt.%, however, defects become more noticeable. The increase of CF content from 0 wt.% to 10 wt.% led to an increased UTS with a maximum UTS of 9.23 MPa and a maximum Young’s modulus of 310 MPa was seen for 14 wt.% CF + HDPE composite. UTS decreased by further increasing CF content due to the generation of internal defects in the 14 wt.% CF-reinforced HDPE composite. An overall decrease in percentage elongation is found with CF content increasing. As for toughness, the 6 wt.% CF-reinforced HDPE composite exhibits the largest toughness of 7.72 MJ/mm^3^. When CF content ranges from 0 wt.% to 14 wt.%, at a specific temperature, the higher the CF content is, the higher the storage modulus (and loss modulus) will be. This value is in line with the Young’s modulus obtained from the tensile test as expected. The loss tangent values of all parts are below 0.273, indicating that the parts act more elastically than plastically. CFs also improve the thermal stability of HDPE by increasing the decomposition temperature by ~22 °C.

## Figures and Tables

**Table 1:** Carbon fiber specifications.

Parameters	Units	Values/Ranges
Fiber length	μm	50~200
Average fiber diameter	µm	7.2
Density	g/cc	1.75
Linear resistivity	Ω/cm	0.069
Electrical resistivity	Ω/cm	0.0014
Carbon content	%	99

**Table 2:** Process parameters of the FDM process.

Input Variables	Units	Values
Initial layer flow rate	%	100
Initial layer thickness	mm	0.25
Flow rate	%	100
Layer thickness	mm	0.2
Infill density	%	90
Scanning speed	mm/s	20
Build platform temperature	°C	120
Nozzle temperature	°C	260

**Table 3:** A summary of tensile properties.

	UTS (MPa)	Young’s Modulus (MPa)	Percentage Elongation (%)	Toughness (MJ/mm^3^)
HDPE	5.33 ± 0.12	95 ± 5	150 ± 10	7.18 ± 0.30
2 wt.% CF + HDPE	6.61 ± 0.24	145 ± 5	102 ± 6	5.89 ± 0.26
6 wt.% CF + HDPE	9.03 ± 0.31	180 ± 5	97 ± 5	7.72 ± 0.28
10 wt.% CF + HDPE	9.23 ± 0.28	220 ± 5	89 ± 5	7.13 ± 0.18
14 wt.% CF + HDPE	8.97 ± 0.35	310 ± 5	62 ± 3	4.68 ± 0.13

**Table 4:** A summary of dynamic mechanical properties.

	Storage Modulus (MPa)	Loss Modulus (MPa)	Loss Tangent Range
HDPE	149.36	25.81	0.124~0.259
2 wt.% CF + HDPE	157.10	28.58	0.083~0.256
6 wt.% CF + HDPE	183.03	28.96	0.114~0.247
10 wt.% CF + HDPE	207.85	31.97	0.135~0.272
14 wt.% CF + HDPE	313.36	46.72	0.125~0.273

## Data Availability

The data presented in this study are available on request from the corresponding author.
